# Netrin-1 promotes diabetic corneal wound healing through molecular mechanisms mediated via the adenosine 2B receptor

**DOI:** 10.1038/s41598-018-24506-9

**Published:** 2018-04-16

**Authors:** Yangyang Zhang, Peng Chen, Guohu Di, Xia Qi, Qingjun Zhou, Hua Gao

**Affiliations:** 1 School of Medicine and Life Sciences, University of Jinan-Shandong Academy of Medical Sciences, Jinan, China; 2 State Key Laboratory Cultivation Base, Shandong Provincial Key Laboratory of Ophthalmology, Shandong Eye Institute, Shandong Academy of Medical Sciences, Qingdao, China

## Abstract

Netrins are secreted chemoattractants with the roles in axon guidance, cell migration and epithelial plasticity. In the present study, we investigated the roles of netrin-1 in the regulation of corneal epithelial wound healing, inflammation response and nerve fiber regeneration in diabetic mice and cultured corneal epithelial cells. In diabetic mice, the expression of netrin-1 was decreased when compared with that of normal mice. Furthermore, high glucose blocked the wounding-induced up-regulation of netrin-1 expression in corneal epithelial cells. Exogenous netrin-1 promoted the corneal epithelial wound healing in diabetic mice, and facilitated the proliferation and migration by reactivating the phosphorylation of ERK and EGFR in high-glucose treated corneal epithelial cells. Moreover, netrin-1 decreased the neutrophil infiltration and promoted M2 macrophage transition, accompanied with the attenuated expression of pro-inflammatory factors in diabetic mouse corneal epithelium. The promotions of netrin-1 on corneal epithelial wound healing and inflammation resolution were mediated at least through the adenosine 2B receptor. In addition, netrin-1 promoted the regeneration of corneal nerve fibers that was impaired in diabetic mice. Taken together, netrin-1 regulates corneal epithelial wound healing, inflammation response and nerve fiber regeneration in diabetic mice, indicating the potential application for the therapy of diabetic keratopathy.

## Introduction

The corneal epithelium is subjected continuously to physical, chemical, and biological insults, often resulting in a wound and loss of barrier functions. Normally corneal epithelium responds rapidly to injury, which involves cell migration, cell proliferation, re-stratification, as well as matrix deposition and tissue remodeling. Proper healing of corneal wounds is vital for maintaining a clear, healthy cornea and for preserving vision. Corneal keratopathy occurs in more than 70% of diabetic patients which manifested as impaired corneal sensation, persistent epithelial defects, and recurrent erosion^1,2^. Corneal nerves play an important role in the regulation of the blink reflex, epithelium homeostasis, tear production and secretion^3,4^. The sensory nerve fibers in diabetic patients with peripheral neuropathy undergo the earliest damage in diabetes. The attenuation of corneal innervation, caused by diabetic mellitus always produces impaired corneal sensation, chronic inflammation, delayed corneal epithelial wound healing, and even persistent defect^5,6^. Neurotrophic deficits may play a major role in the pathogenesis of diabetic keratopathy, the most recognized diabetic complication in cornea, as the corneal nerve fibers are reported to exert important trophic influences and contribute to the maintenance of corneal epithelium homeostasis^7^.

Netrins are a conserved family of laminin-related secreted proteins with multiple functions in cell migration and axon guidance during embryogenesis. Among these, netrin-1 was initially discovered as the main attractive cue for commissural axon guidance by acting through its receptor deleted in colorectal cancer (DCC)^8^. Several netrin-1 receptors have been reported previously, including DCC and neogenin (DCC family), uncoordinated family member 5A-D (UNC5 families), Down syndrome cell adhesion molecule (DSCAM), and α6β4 and α3β1 integrins^9–11^. Recently, the adenosine 2B receptor (A2BAR) has also been identified as the receptor of netrin-1^12^. According to previous descriptions, netrin-1, mediated through different receptors, may regulate different signaling pathways and play distinct roles in physiological and pathophysiological conditions^13^. Other than the function of axon guidance, netrin-1 can also protect the kidney against ischemia-reperfusion injury, regulate angiogenesis, and even promote tumor growth^14–17^. In addition, netrin-1 was found recently to possess an anti-inflammatory capacity in lung injury and inflammatory peritonitis^18,19^. In cornea, a previous study has confirmed that netrin-1 can dampen alkali burn-induced inflammation and neovascularization^20^.

Based on the multiple functions of netrin-1 and the characteristics of diabetic corneal pathogenesis, we hypothesized that netrin-1 may assume a potential use for the treatment of diabetic keratopathy. To address this hypothesis, we studied the regulation and mechanism of netrin-1 on corneal epithelial wound healing, inflammatory response, and nerve fiber regeneration by using type 1 diabetic mice and high glucose-treated corneal epithelial cells.

## Results

### Hyperglycemia causes decreased netrin-1 expression in corneal epithelium

**Figure 1:**
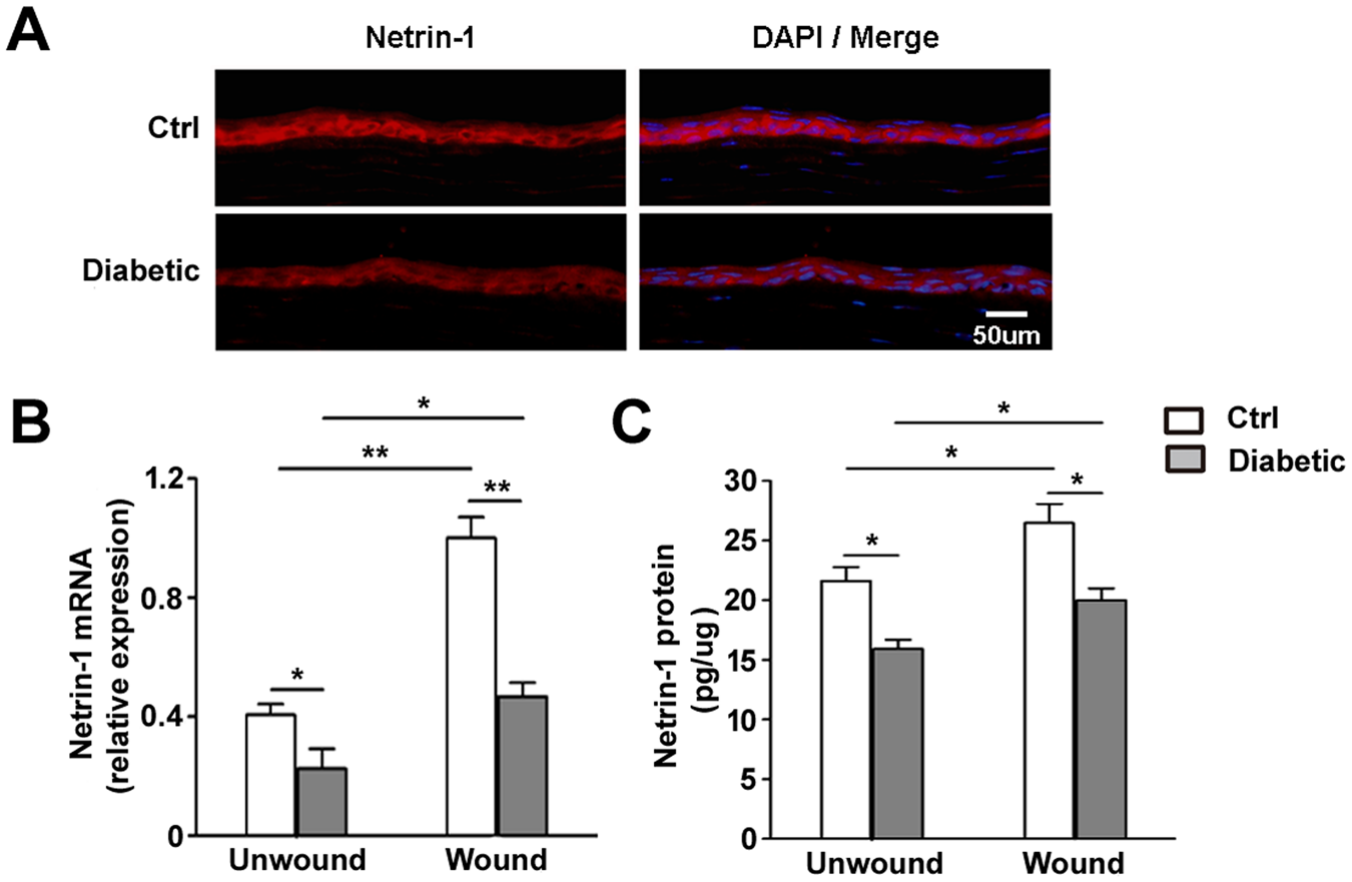
Hyperglycemia downregulates netrin-1 expression in corneal epithelium. Normal and diabetic mouse corneal epithelium was collected before (as unwound group) or 48 h after epithelial scrape (as wound group). Netrin-1 expression was examined by using immunofluorescence staining (**A**, 3 mice per group), RT-qPCR (**B**, 9 mice per group) and ELISA (**C**, 9 mice per group) with the age-matched normal mice as control. *p < 0.05, **p < 0.01.

To examine the expression of netrin-1, mouse cornea and epithelium were collected and analyzed by using immunofluorescence staining, RT-qPCR and ELISA. The immunofluorescence staining of netrin-1 in normal and diabetic unwound mice corneal sections showed that the expression of netrin-1 in diabetic mouse was decreased (Fig. 1A). In unwounded diabetic mice, the mRNA transcripts of netrin-1 were significantly downregulated (Fig. 1B) when compared with that of control mice. Moreover, the netrin-1 concentration was reduced in diabetic mice corneal epithelium compared to that of control mice (Fig. 1C). Furthermore, the mRNA and protein levels of netrin-1 were downregulated in regenerating corneal epithelium of diabetic mice compared to those levels in control mice (Ctrl vs. Diabetic in Fig. 1 B,C). The results suggest that the netrin-1 expression was decreased and the function of netrin-1 upregulated stressed by wounding was impaired in diabetic mice.

### Netrin-1 promotes corneal epithelial wound healing in diabetic mice

**Figure 2:**
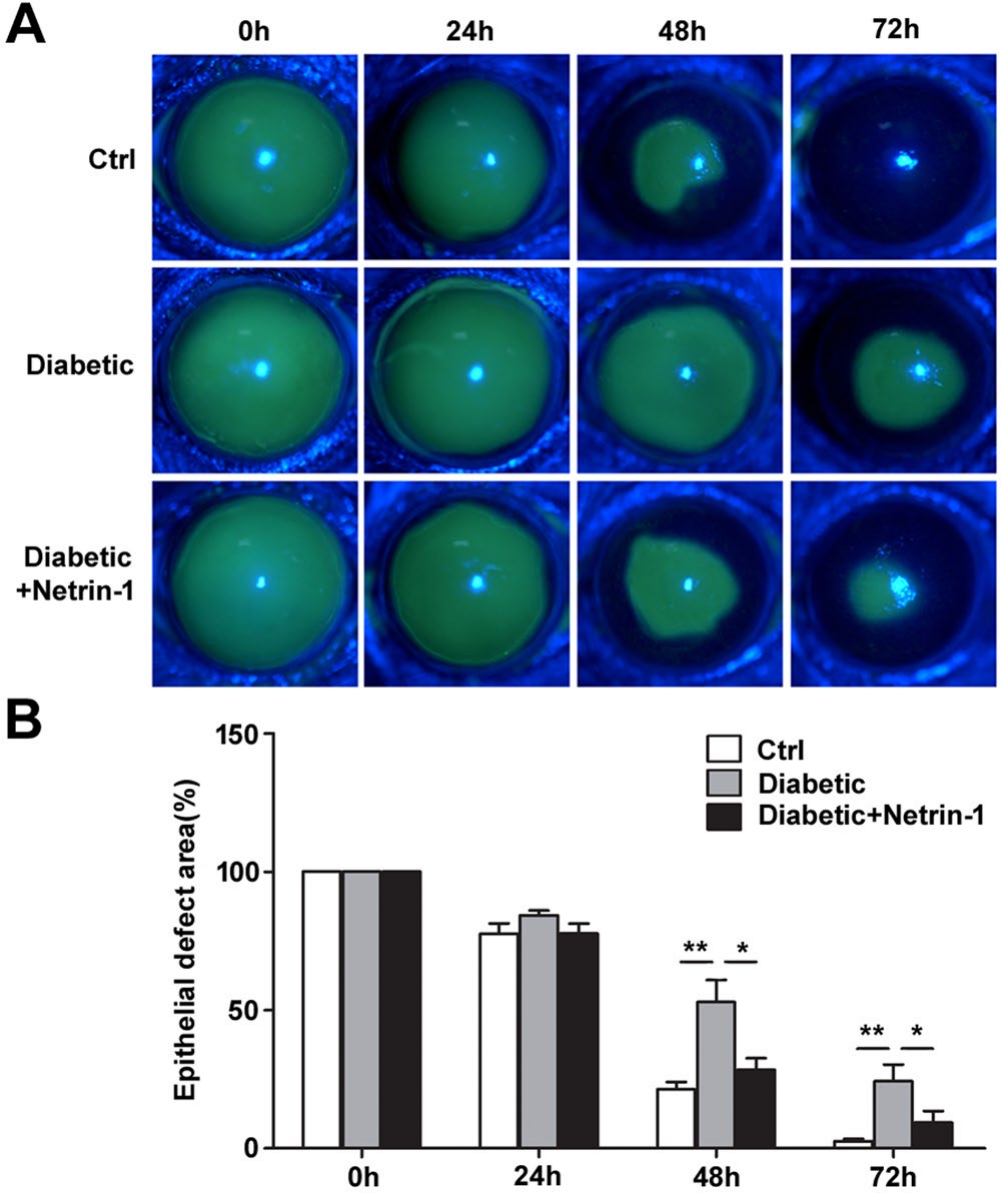
Netrin-1 promotes corneal epithelial wound healing in diabetic mice. Corneal epithelium was scraped in control and diabetic mice with or without subconjunctival injection of netrin-1 (50 ng per eye). The corneal epithelial wound defect was stained with fluorescein sodium at 24, 48, and 72 h after epithelial scrape (**A**, 8 mice per group). The histogram of residual epithelial defect is presented as the percentage of the original wound area (**B**). *p < 0.05, **p < 0.01.

To investigate the effects of netrin-1 on diabetic corneal epithelial wound healing, the entire corneal epithelium was scraped in diabetic mice and their age-matched control mice. The diabetic mice were subsequently treated with netrin-1via a single subconjunctival injection. The corneal epithelial remaining wounds showed a significant difference at 48 and 72 h after epithelial scrape (Fig. 2A,B). The remaining wounds of corneal epithelium in netrin-1-treated diabetic mice (48 h, 28.37%±9.57%; 72 h, 9.30%±9.44%) was significantly smaller than that of diabetic mice (48 h, 52.87%±17.98%; 72 h, 24.28%±13.56%), although still larger than that of control mice (48 h, 21.33%±5.60%; 72 h, 2.58%±1.92%). Moreover, topical applications of netrin-1 also showed similar promotion of corneal epithelial wound healing in diabetic mice (Figure S1). In addition, exogenous netrin-1 also promoted the normal mice corneal epithelial wound healing (data not shown).

### Netrin-1 promotes migration, proliferation, and ERK and EGFR phosphorylation of corneal epithelial cells impaired by high glucose

**Figure 3:**
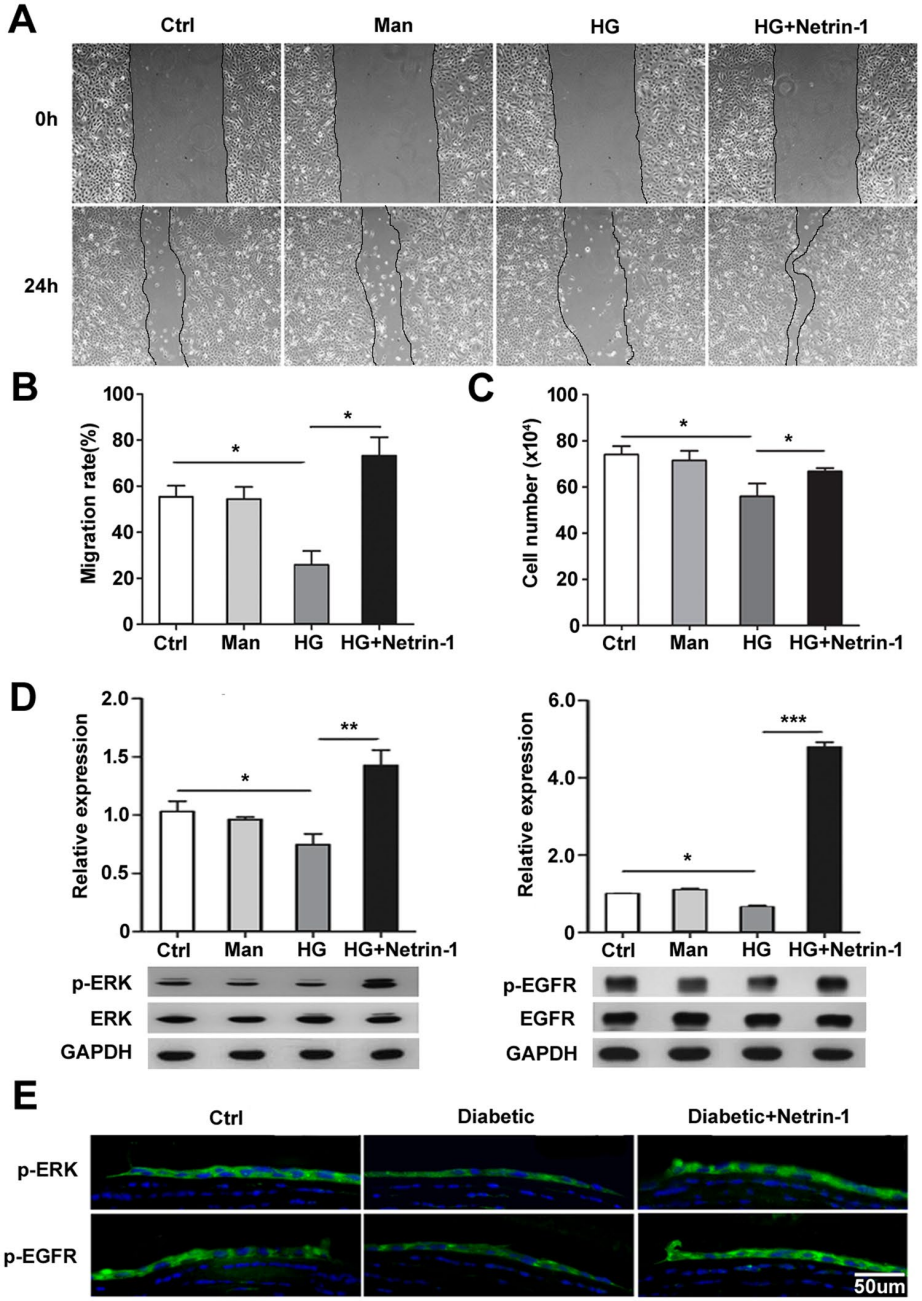
Netrin-1 promotes the migration, proliferation, and ERK and EGFR phosphorylation of corneal epithelial cells impaired by hyperglycemia. Confluent mouse corneal epithelial cells were wounded after incubation with 5 mM glucose, 30 mM mannose or 30 mM glucose. Cell migration was observed with or without 50 ng/mL netrin-1 treatment for 24 h (**A**). Migration rate was analyzed and represented as the percentage of primary wounding area (**B**, n = 3 per group). Cell proliferation was measured by cell counting after 3 days of netrin-1 treatment (**C**, n = 3 per group). The phosphorylated levels of ERK and EGFR were analyzed by Western blotting after 3 days incubation until monolayer formation. We administrated netrin-1 (50ng/ml) and after 8 or 15 minutes we collected the cells and then detected the p-ERK or p-EGFR expression by western blotting. (**D**, n = 4 per group). The expression of p-ERK/p-EGFR were also confirmed by immunofluorescence staining in the netrin-1 injected diabetic mouse corneal epithelium 48 h after injury (**E**, 3 mice per group). *p < 0.05, **p < 0.01, ***p < 0.001.

To determine the impact of netrin-1 on corneal epithelial wound healing *in vitro*, mouse corneal epithelial cells were incubated with high glucose, with an equal concentration of mannose as the osmotic control, until monolayer formation. To detect the migration of TKE-2 cell incubated with high glucose, the corneal epithelial monolayer was wounded and incubated with or without netrin-1 (50 ng/mL) for 24 h. The results showed that corneal epithelial wound closure was significantly delayed by high glucose treatment while netrin-1 promoted the migration capacity of high glucose-treated cells to the same level of normal cells (Fig. 3A,B). To evaluate the proliferation of netrin-1, we inoculated and cultured 10^5^ TKE-2 cells with normal and high glucose medium with/without netrin-1. After 3 days, we collected and amounted the cells, the results showed that netrin-1 improved the proliferation potential of corneal epithelial cells that was impaired by high glucose treatment (Fig. 3C). To elucidate the mechanism underlying the promotion of netrin-1 on corneal epithelial wound healing, we investigated the effects of netrin-1 on the upregulation of EGF expression and activation of ERK and EGFR that were impaired in diabetic corneal epithelium. The immunofluorescence staining of EGF in mice corneal epithelium showed that the immunofluorescence luminance of EGF in diabetic group were weaker than normal group and netrin-1 treatment can effectively increase the expression of EGF in diabetic mice (Figure S2). The results of western blotting showed that high glucose-cultured cells exhibited reduced activation of ERK and EGFR while netrin-1 significantly increased the phosphorylation levels of ERK and EGFR to even above that of control cells (Fig. 3D), which was also confirmed by immunofluorescence staining in the netrin-1-injected diabetic mouse corneal epithelium 48 h after injury (Fig. 3E).

### Netrin-1 promotes inflammation regression in diabetic cornea

**Figure 4:**
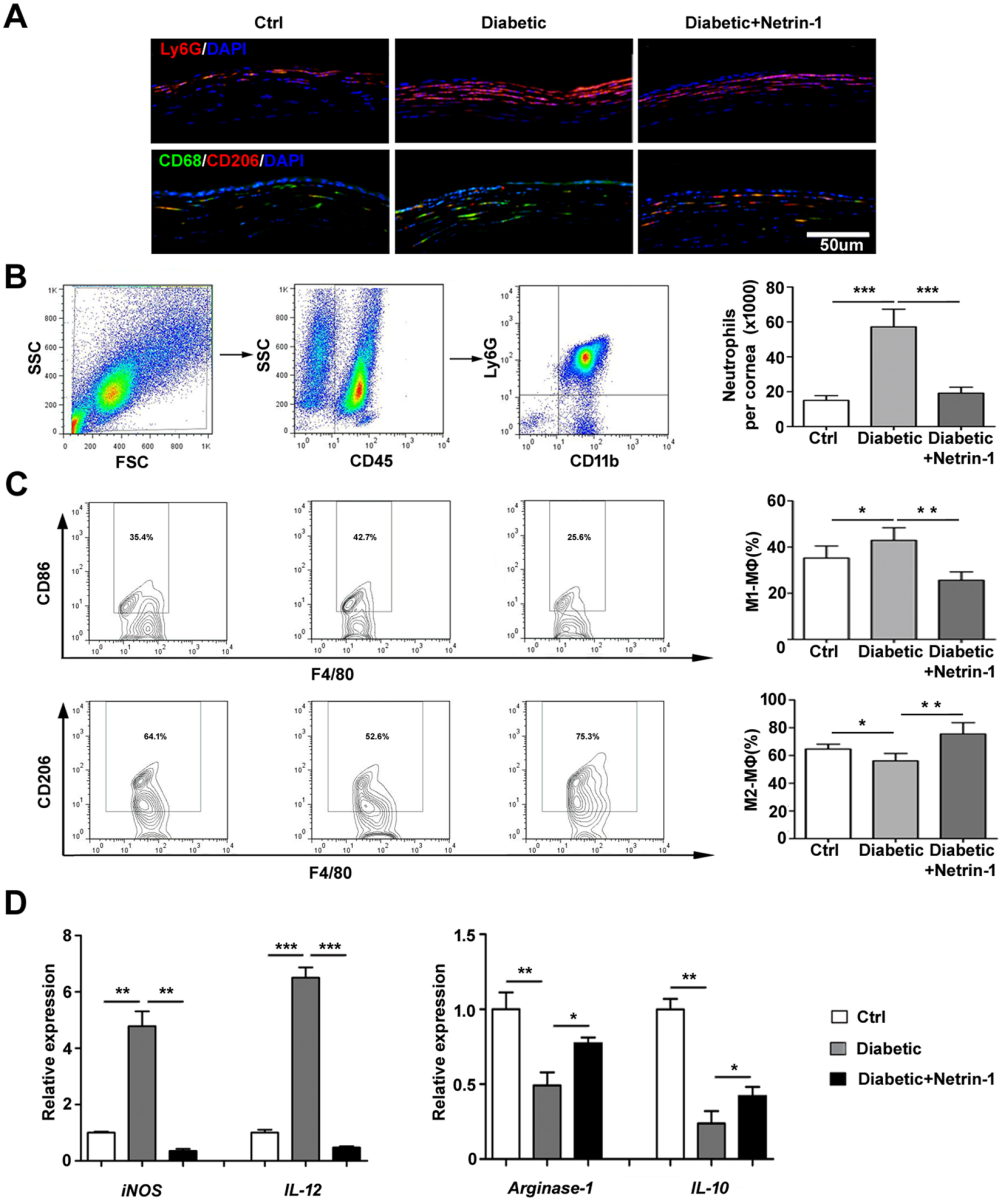
Netrin-1 promotes inflammation resolution in diabetic corneal wound healing. Corneal epithelium was scraped in control and diabetic mice with or without subconjunctival injection of netrin-1 (50 ng per eye). Mouse cornea was collected and immunofluorescent stained with neutrophil marker Ly6G at 48 h and M2 macrophage markers CD68 (green fluorescence) and CD206 (red fluorescence) at 72 h after epithelial scrape (**A**, 3 mice per group). Flow cytometry was performed to quantify the number and percentage of neutrophils (CD45 + CD11b + Ly6G+) (**B**, 12 mice per group) at 48-hour post-wounded, M1 macrophages (CD45 + F4/80 + CD86+) and M2 macrophages (CD45 + F4/80 + CD206+) at 72-hour post-wounded. (**C**, 12 mice per group at each detection) mRNA expression levels of *iNOS* and *IL-12* (48 h after epithelial scrape), *arginase-1* and *IL-10* (72 h after epithelial scrape) were analyzed by RT-qPCR from the control and diabetic mouse corneas (**D**, 6 mice per group); *p < 0.05, **p < 0.01, ***p < 0.001.

Persistent low-grade inflammation is known to be a large cause of diabetic complications. To explore the possible anti-inflammatory effects of netrin-1, recombinant netrin was used to treat diabetic, wounded corneas and its effects on the infiltrations of neutrophils, macrophages were examined at 48 and 72 h after the corneal epithelial scrape. Representative staining and flow cytometry were shown in Fig. 4. Netrin-1 decreased the infiltration of neutrophils (Ly6G+, 48 h, Fig. 4A,B) and increased the number of M2 macrophages (CD68+/ F4/80+CD206+, 72 h, Fig. 4A,C) in diabetic cornea compared to that of diabetic cornea without netrin-1 treatment. Moreover, the mRNA levels of *iNOS* and *IL-12* (markers of M1 macrophages) were significantly decreased with the treatment of netrin-1 in diabetic cornea at 48 h after the corneal epithelial scrape, while the expressions of *arginase-1* and *IL-10* (markers of M2 macrophages) were increased with the netrin-1 treatment at 72 h after the corneal epithelium scrape (Fig. 4D). These results suggest exogenous netrin-1 promotes the resolution of inflammation during diabetic corneal wound healing.

### Netrin-1 promotes diabetic corneal epithelial wound healing through Adenosine 2B receptor

**Figure 5:**
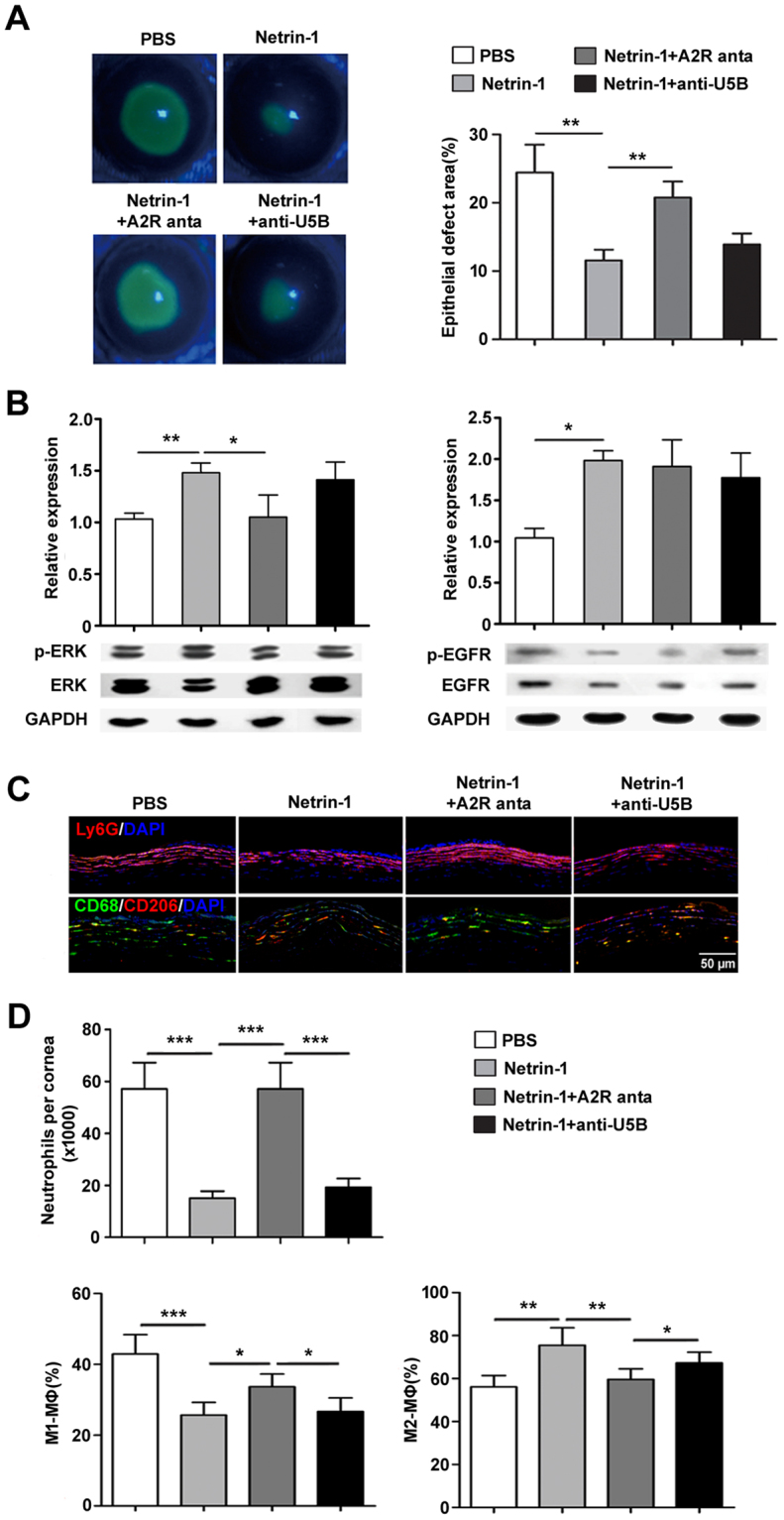
The A2BAR receptor mediates the promotion of netrin-1 on diabetic corneal wound healing. A2BAR receptor-specific antagonist or anti-UNC5B blocking antibody was injected subconjunctivally at 24 h before and 0, 24, and 48 h after the scrape of corneal epithelium in netrin-1 treated diabetic mice. Corneal epithelium defect was stained with fluorescein sodium and presented as the percentage of the original wound at 72 h after epithelial scraped (**A**, 3 mice per group). Activation of ERK and EGFR in corneal epithelium were analyzed by western blotting at 48 h after epithelial scraped (**B**, 3 mice per group). Neutrophils infiltration to the cornea and the cell numbers were examined by immunofluorescence staining (**C**, 3 mice per group) and flow cytometry (**D**, 12 mice per group) at 48 h after epithelial scraped. Macrophages infiltration to the cornea and the cell numbers were examined by immunofluorescence staining (C, 3 mice per group) and flow cytometry (**D**, 12 mice per group of each detection) at 72 h after epithelial scraped. A2R anta: A2BAR antagonist; anti-U5B: anti-UNC5B blocking antibody. *p < 0.05, **p < 0.01, ***p < 0.001.

In our preliminary experiment, we found that Adenosine 2B receptor and *UNC5B* receptor were significantly upregulated when cultured mouse corneal epithelial cells were either wounded or treated with netrin-1 (Figure S3). To further determine whether *A2BAR* and/or *UNC5B* receptors mediate the improvement of netrin-1 on diabetic corneal wound healing, the *A2BAR* receptor-specific antagonist PSB1115 or anti-UNC5B blocking antibody was injected subconjunctivally in netrin-1-treated diabetic mice. The results showed that the *A2BAR* antagonist significantly attenuated the promotion of netrin-1 on diabetic corneal epithelial wound healing, with 20.77%±2.35% epithelial defect in antagonist-injected netrin-1-treated mice compared to only 11.59%±1.55% defect in mice treated with netrin-1 alone and to 24.44%±4.09% defect in untreated diabetic mice at 72 h (Fig. 5A). Moreover, upregulation of the phosphorylated ERK level by netrin-1 was also blocked by the pretreatment of *A2BAR* antagonist in diabetic corneal epithelium, while the phosphorylated EGFR level was not changed with the treatment of *A2BAR* antagonist (Fig. 5B). However, the injection of *UNC5B* blocking antibody showed no effect on wound healing or the levels of phosphorylated ERK and EGFR in the corneal epithelium of diabetic mice (Fig. 5B). In addition, treatment with *A2BAR* antagonist but not with *UNC5B* blocking antibody abolished the regulation of netrin-1 on the infiltration of neutrophils and the phenotype transition of macrophages in diabetic corneal wound healing (Fig. 5C,D). The results suggest that the adenosine 2B receptor is involved in the mediation of netrin-1 on the promotion of diabetic corneal wound healing.

### Netrin-1 enhances nerve fibers regeneration in diabetic cornea

**Figure 6:**
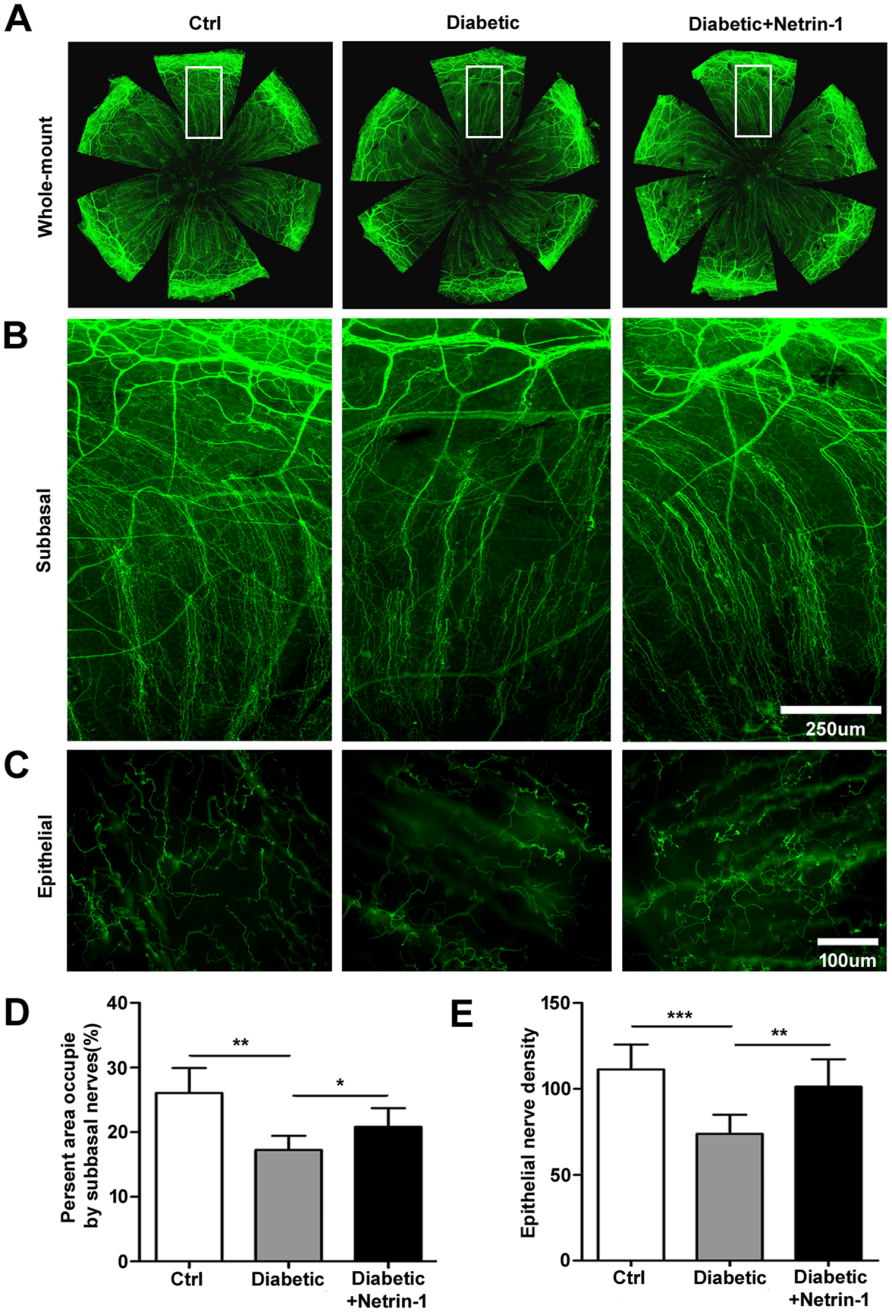
Netrin-1 promotes the corneal nerve regeneration in diabetic mice. Netrin-1 (50 ng per eye) was subconjunctivally injection after epithelial scrape in diabetic mice. Overall corneal nerves (**A**), subbasal (**B**) and intraepithelial nerve fibers (**C**) were monitored at 14 days after epithelial scrape. Subbasal and intraepithelial nerve fiber density (**D** and **E**, 4 mice per group) were measured and quantified by Image J software, *p < 0.05, **p < 0.01, ***p < 0.001.

To elucidate whether netrin-1 enhances corneal nerve fiber regeneration in diabetic mice, 50ng netrin-1(5 *μ*L/eye) or PBS was injected twice a week subconjunctivally after the scrape of central corneal epithelium. After 14 days, the corneas were collected and whole-mount stained with β3-tubulin to mark the nerve fibers. The corneal subbasal nerve fibers and intraepithelial nerves^21^ density in diabetic mice showed apparent decline compared with control mice, whereas mouse corneas with netrin-1 injection exhibited relatively densely in the subbasal nerve fibers and intraepithelial nerves (Fig. 6A,B,C). According to the analysis of present area occupied by subbasal nerves and intraepithelial nerves density, there was a significant difference in diabetic and netrin-1 injection mice (Fig. 6D,E)

## Discussion

As one of the most densely innervated tissues, the cornea contains abundant nerve fibers that branch from the ophthalmic trigeminal ganglion. A number of neurotrophic factors and neuropeptide have previously been detected in the corneal epithelium, among which the pigment epithelium-derived factor (PEDF)^1^, nerve growth factor (NGF), glial cell-derived neurotrophic factor (GDNF)^22^ and substance P^2^ were involved in the regulation of corneal innervation in the corneal wound healing. As a traditional guidance cue for axon growth in the central and peripheral nervous system, previous study has confirmed the expression of netrin-1 in adult mouse corneal epithelium^20^. Herein this study, we found that netrin-1 expression was significantly decreased in both mRNA and protein levels in the corneal epithelium of diabetic mice when compared with normal mice. Furthermore, high glucose blocked the upregulation of netrin-1 expression induced by wounding in cultured corneal epithelial cells. Exogenous netrin-1 promoted corneal epithelial wound healing of normal and diabetic mice, and the proliferation and migration of high glucose-treated corneal epithelial cells.

Netrin-1 not only functions as an axon guidance factor in nervous system but also has many functions in non-nervous system, such as regulates the adhesion and migration of a variety of epithelial cells, including pancreatic epithelial cells^10^, mammary epithelial cells^23^, and renal proximal tubular epithelial cells^24^. In diabetic keratopathy, one of the most known characteristics is delayed corneal re-epithelialization caused by the impaired activation of the EGFR-, ERK-, and PI3K/AKT-signaling pathways^25^. Here, we demonstrated that exogenous netrin-1 facilitated diabetic corneal epithelial wound healing by upregulated of EGF expression and afterwards reactivating the phosphorylation of EGFR and ERK to promote the migration and proliferation of high glucose-treated corneal epithelial cells.

A2BAR and UNC5B are two classical receptors of netrin-1. Previous studies have shown that the potent effect of netrin-1 on inhibition of inflammatory response by reducing neutrophil infiltration in some non-ocular tissues is variously dependent on receptor A2BAR^18,19^ or UNC5B^26^ Moreover, A2BAR and UNC5B were the most two significant-decreased receptors in the expression level of diabetic corneal epithelium than that of control epithelium *in vivo* and significantly up-regulated after the mouse corneal epithelial cells were wounded in normal conditions. Combined with our preliminary results and previous descriptions in cornea, we subsequently investigated their roles in the promotion of netrin-1 in diabetic corneal wound healing by using the antagonist or blocking antibody. The *in vivo* application showed the A2BAR antagonist, but not the UNC5B blocking antibody, reversed the promotion of netrin-1 in diabetic corneal wound healing, which suggests that at least the A2BAR mediates the promotion of netrin-1 on diabetic corneal epithelial wound healing. However, considering to the possible different diffusing capacity caused by different molecular weight between antagonist and blocking antibody, the exact mechanism of netrin-1 on the promotion of diabetic corneal wound healing needs to be further investigated, including the possible involvement of other reported receptors^27^.

In acute normal wound healing, inflammation protects against infection and injury but must be timely resolved to prevent unwarranted tissue damage. The resolution of inflammation is accomplished by blunting neutrophil infiltration, decreasing pro-inflammatory factor production, promoting the macrophage phagocytosis of apoptotic cells and microbes, and stimulating the clearance of phagocytes to enable a return to homeostasis^23^. Moreover, the excessive inflammation and impaired inflammation resolution, characterized by excessive infiltration of neutrophils and transition impairment of M1- to M2-macrophage phenotype, has been demonstrated to contribute to impaired wound healing in diabetic mellitus^24^. In the present study, netrin-1 attenuated the infiltration of neutrophils and decreased the expression levels of pro-inflammatory factors in corneal wound healing of diabetic mice. In addition, netrin-1 also promoted the generation of M2-macrophages, which was impaired in the corneal wound healing of diabetic mice. The results were consistent with the previous descriptions of the anti-inflammatory activity of netrin-1 in diabetic nephropathy^28–31^, acute colitis^28,29,31^, pancreatitis^30^, lung injury^18^, and cornea by inhibiting neutrophil infiltration and inducing the M2 polarization of macrophages^20,32^. More interesting, when treated with A2BAR antagonist, rather than UNC5B blocking antibody, the induced the M2 polarization of macrophages was reversed, suggesting that the promotion of netrin-1 on M2 macrophage transition was signaling through the A2BAR, but not UNC5B.

The decreased corneal sensation and corneal nerve fiber density are the typical characteristics of diabetic keratopathy^33,34^. The dysfunction of corneal innervation is critical for the impairment of corneal re-epithelialization wound healing in diabetic mellitus^35^. In the present study, topical netrin-1 promoted the regeneration of diabetic mouse corneal nerve fibers *in vivo*. These findings suggest that netrin-1 expressed in corneal epithelium maintains the homeostasis of corneal epithelial innervation while the reduction of netrin-1 in corneal epithelium caused by hyperglycemia may be involved in the regression of corneal nerve fibers that is found in diabetic mellitus.

In summary, we show that netrin-1 regulates corneal epithelial wound healing, inflammation response, and nerve fiber regeneration in diabetic mice. The multiple function of netrin-1 provides a candidate for treatment of persistent corneal epithelial defects in patients with diabetic mellitus.

## Methods

### Animals

Six-8weeks old male C57BL/6 mice were purchased from the Beijing Pharmacology Institute (Chinese Academy of Medical Sciences, Beijing, China). All animal experiments were approved by the Ethics Committee of Shandong Eye Institute and carried out in accordance with the Association for Research in Vision and Ophthalmology (ARVO) Statement for the Use of Animals in Ophthalmic and Vision Research. The mice underwent induction of type 1 diabetes mellitus with intraperitoneal streptozotocin (STZ; Sigma, St. Louis, MO) injections as our previous description^2^. In the present study, diabetic mice were used 12 weeks after the final STZ injection, with the blood glucose level above 350 mg/dL. We have used at least 114 normal mice and 300 diabetic mice in the experiments we showed.

### Corneal epithelial wound healing

Mouse entire corneal epithelium, including the limbal region, was marked with 3 mm trephine and subsequently scraped with a corneal rust ring remover (Alger Co, Lago Vista, TX) in anesthetized normal and diabetic mice. After the corneal epithelium scraped, diabetic mice were injected subconjunctivally with 50 ng netrin-1 (5 *μ*L/eye, R&D Systems, Minneapolis, MN) or equal phosphate buffer saline (PBS) as the vehicle control. For topical netrin-1 application, netrin-1 (50 ng in 2.5 *μ*L PBS) was dropped onto the corneal surface by a 10 *μ*L tip four times daily per eye for 3 days. For netrin-1 receptor inhibition, A2BAR antagonist PSB1115 (2.5 *μ*g in 5 *μ*L PBS, R&D Systems) or UNC5B receptor-blocking antibody (5 *μ*g in 5 *μ*L PBS, R&D Systems) was injected subconjunctivally at 24 h before and 0, 24, and 48 h after the scrape of corneal epithelium. Ofloxacin eye drops were applied to all mice to avoid infection. After 24, 48, and 72 h, corneal epithelium defects were visualized by staining with fluorescein sodium and photographed under slit lamp microscope (BQ900; Haag-Streit, Bern, Switzerland). The stained area on the residual epithelial defect was analyzed by using Image J software (National Institutes of Health, Bethesda, MD) with the original epithelial defect area as 100%.

### Cell culture and treatment

The mouse corneal epithelial cell line TKE2, provided by Tetsuya Kawakita of Keio University (Tokyo, Japan), was cultured in keratinocyte serum-free medium (KSFM; Invitrogen, Carlsbad, CA)^36^. For the analysis of ERK and EGFR phosphorylation, the cells were starved overnight in bovine pituitary extract (BPE)-free KSFM (for ERK analysis) or BPE and EGF-free KSFM (for EGFR analysis) and subsequently incubated with 30 mM glucose or mannose (as osmotic control) in the presence of 10~100 ng/mL netrin-1.

### Cell migration and proliferation analysis

For migration analysis, the cells were incubated with high glucose until confluence, wounded with a micropipette tip, and incubated with or without 50 ng/mL netrin-1 for 24 h. Cells were photographed, and the wound closure was measured by Image J software. To determine the effect of netrin-1 on the proliferation of corneal epithelial cells, the TKE2 cells were starved overnight in BPE-free KSFM and subsequently incubated with 20 ng/mL netrin-1 for 3 days. The cell number was measured with an automated cell counter (Invitrogen Life Technologies, Paisley, UK).

### Immunofluorescence and corneal whole mount staining

Frozen sections (8 *μ*m) were fixed with 4% paraformaldehyde, permeabilized with 0.1% Triton X-100, and blocked with 10% bovine serum albumin at room temperature. The samples were incubated with primary antibodies overnight at 4 °C and subsequently with corresponding secondary antibodies incubate for 1 h at room temperature. The information for primary and secondary antibodies are showed in Table 2. All samples were counterstained with 4’,6-diamidino-2-phenylindole (DAPI, Sigma), and images were captured with an Eclipse TE2000-U microscope (Nikon, Tokyo, Japan). Corneal whole-mount immunofluorescence staining was performed as we and other researchers described^21,37,38^. Mouse eyeballs were collected and fixed in Zamboni’s fixative for 1 h, then the cornea was dissected around the scleral-limbal region and blocked by PBS with 0.1% Triton X-100, 2% goat serum, and 2% bovine serum albumin for 2 h, and subsequently incubated in the same incubation buffer with Alexa Fluor® 488 conjugated neuronal class III β-tubulin mouse monoclonal antibody overnight at 4 °C. After washing for 5 times, the flat mounts were examined under an Eclipse TE2000-U microscope. The quantification of corneal innervation was calculated as the percentage of area positive for β-tubulin staining as previously described^39–41^.

### Reverse-transcription quantitative polymerase chain reaction

For reverse-transcription (RT) quantitative polymerase chain reaction (qPCR), total RNA was extracted from the whole cornea (for detection of pro-inflammatory factor expression, 2 corneas were pooled as a sample) or corneal epithelium (for detection of netrin-1 and receptors expression, 2 corneas were pooled as a sample) or cultured TKE2 cells, using Nucleospin RNA kits (Macherey-Nagel, Düren, Germany) according to the instructions supplied by manufacturer. Corneal epithelium was collected by scraper under the microscope after the mice were executed. Complementary DNAs were synthesized using the Primescript™ First-Strand cDNA Synthesis kit (TaKaRa, Dalian, China). Real-time PCR was performed using SYBR® Green PCR reagents and the Applied Biosystems 7500 Real Time PCR System (Applied Biosystems, Foster City, CA). The specific primers used are listed in Table 1. The cycling condition was 10 secs at 95 °C followed by 45 two-step cycles (15 sec at 95 °C and 1 min at 60 °C). The quantification data were analyzed with Sequence Detection System software (Applied Biosystems) by using GAPDH as the internal control.

**Table 1:** Primer sequences for RT-qPCR.

Gene	Accession number	Forward primer	Reverse primer
Netrin-1	NM_008744.2	TGCAACCGATGTGCCAAA	TTGGAGGCCTTGCAATAGGA
UNC5A	NM_153131.3	GGAGTACTGGTGCCAGTGTG	ATAGGCAATCCGGATGTAGG
UNC5B	NM_029770.2	CCAAGCCTTTGCTCTTTAAGGA	GCTCTCTGGCTGCCATTCC
UNC5C	NM_009472.3	CTGCACCTTCACTCTGGAAA	AGATCTGCCCTTCTCCTTCA
UNC5D	NM_153135.3	AAGTGCATGCTTGGAAACAG	TGGCAATCTGCTTTCACTTC
A2BAR	NM_007413.4	CCGCTCAGGTATAAAGGTTTGG	CCAGGAACGGAGTCAATCCA
iNOS	NM_010927.3	TGTCTGCAGCACTTGGATCAG	AAACTTCGGAAGGGAGCAATG
Arginase-1	NM_007482.3	TGGGTGACTCCCTGCATATCT	TTCCATCACCTTGCCAATCC
IL-12	NM_008351.2	GGGACCAAACCAGCACATTG	TACCAAGGCACAGGGTCATCA
IL-10	NM_010548.2	TGCTAACCGACTCCTTAATGCA	TTCTCACCCAGGGAATTCAAA

### Western blotting analysis

Total protein was extracted from lysed samples of the mouse corneal epithelium (3 corneas were pooled as a sample) or cultured TKE2 cells and run on 10% SDS-PAGE gels before the transfer to a PVDF membrane (Millipore, Billerica, MA). The samples were blocked with 5% nonfat dry milk and incubated with primary antibodies overnight at 4 °C and subsequently with horseradish peroxidase-conjugated secondary antibody (Amersham Biosciences, Piscataway, NJ) for 1 h at room temperature. The information for primary antibodies are showed in Table 2. Finally, the blots were visualized via enzyme-linked chemiluminescence by using the ECL kit (Pierce Biotechnology, Rockford, IL) and quantified by using Image J software.

**Table 2:** Antibodies for immunofluorescent staining, flow cytometry and Western blots.

Primary antibody	Dilution concentration	Supplier	Code
p-EGFR	WB(1/1000) IF(1/200)	Epitomics	1727-1
EGFR	WB(1/1000)	Epitomics	1902-1
p-ERK	WB(1/1000) IF(1/200)	Cell Signaling	4370S
ERK	WB(1/1000)	Santa Cruz	sc-145
EGF	IF(1/200)	Abcam	ab184265
Ly6G-PE	IF(1/200) FC(1/200)	Biolegend	127608
CD206-PE	IF(1/200)	Biolegend	141705
CD68-FITC	IF(1/200)	Biolegend	137006
CD11b-FITC	FC(1/200)	Biolegend	101205
CD45-PE-cy5	FC(1/200)	Biolegend	103109
CD86-PE	FC(1/200)	Biolegend	105007
F4/80-FITC	FC(1/200)	Biolegend	123107
III β-tubulin	IF(1/200)	Merck-Millipore	AB15708A4
Alexa Fluor 488 donkey anti-rabbit IgG	IF(1/200)	Life technologies	A21206
Alexa Fluor 594 donkey anti-rabbit IgG	IF(1/200)	Life technologies	A21207

WB: Western-blotting; IF: Immunofluorescence; FC: Flow cytometry.

### Enzyme-linked immunosorbent assay

Total protein was extracted from the intact or regenerating corneal epithelium (48 h after the scrape) of three eyes of normal or diabetic mice. All samples were centrifuged, and the supernatants were subjected to quantitative sandwich immunoassay with the enzyme-linked immunosorbent assay (ELISA) detection kit for mouse netrin-1 (USCN Life Science, Wuhan, China) according to the manufacturer’s instructions. Absorbance was measured with a microplate reader (Molecular Devices, Sunnyvale, CA) at a wavelength of 450 nm.

### Flow cytometry

Mouse eyeballs were collected and fixed in PBS at 48 h or 72 h after injury. Cornea was dissected around the scleral-limbal region and cut into pieces by corneal scissors. We incubated them in 50 uL Liberase TL (2.5 mg/ml; Sigma) for 30 min at 37 °C with gentle stirring and then undigested tissue removed by screen filters (4 corneas were pooled as a sample). We finally collected cell suspensions and incubated them with fluorescein-conjugated anti-mouse antibodies for 30 min at 4 °C and analyzed by flow cytometry with a Vantage flow cytometer (Becton Dickinson) and FlowJo (Tree Star, Inc.) software. The gate was set on CD45+ population, and further analysis of surface markers was done with in this gate. The primary antibodies used were as follows: CD45-PE/-cy5, CD86-PE-cy5, F4/80-FITC, CD11b- FITC, Ly6G-PE and CD206-PE (Biolegend, San Diego, CA). All experiments were repeated at least 3 times.

### Statistical analysis

Data in this study were representative of at least three different experiments and were presented as the means ± standard deviation. Statistical analysis was performed using SPSS 17.0 software (SPSS, Chicago, IL) and one-way analysis of variance. Differences were considered statistical significance at *p* < 0.05.

## Electronic supplementary material

Supplementary Information
