# Supplementary material for: Netrin-1 promotes diabetic corneal wound healing through molecular mechanisms mediated via the adenosine 2B receptor
Source: Sci Rep. 2018 Apr 16;8:5994. doi: 10.1038/s41598-018-24506-9 (PMC5902612; doi:10.1038/s41598-018-24506-9)
Supplement: Supplementary file 1 — Supplementary Information [file 41598_2018_24506_MOESM1_ESM.pdf]

Netrin-1 promotes diabetic corneal wound healing through molecular mechanisms mediated via the adenosine 2B receptor

Yangyang Zhang[1, 2], Peng Chen[2], Guohu Di[2], Xia Qi[2], Qingjun Zhou[2]*, Hua Gao[2]*

1. School of Medicine and Life Sciences, University of Jinan-Shandong Academy of Medical Sciences，Jinan，China

2. State Key Laboratory Cultivation Base, Shandong Provincial Key Laboratory of Ophthalmology, Shandong Eye Institute, Shandong Academy of Medical Sciences, Qingdao, China.

***Correspondence author:** Hua Gao, M.D., Ph.D. & Qingjun Zhou, Ph.D.

Address: Shandong Eye Institute, 5 Yan'erdao Road, Qingdao, 266071, China.

Email: gaohua100@126.com & qjzhou2000@hotmail.com

Phone: 86-532-8589-9270

Fax: 86-532-8589-1110

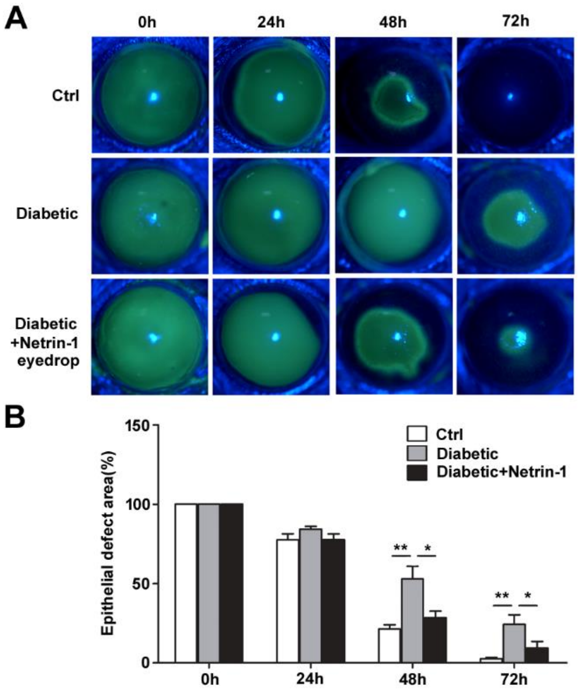

Figure S1. Netrin-1 promotes corneal epithelial wound healing in normal mice. Corneal epithelium was scraped in control mice with subconjunctival injection of equal PBS as vehicle control or netrin-1 (50 ng per eye). The corneal epithelial wound defect was stained with fluorescein sodium at 24, 48, and 72 h after epithelial scrape (A, 8 mice per group). The histogram of residual epithelial defect is presented as the percentage of the original wound area (B). *p<0.05.

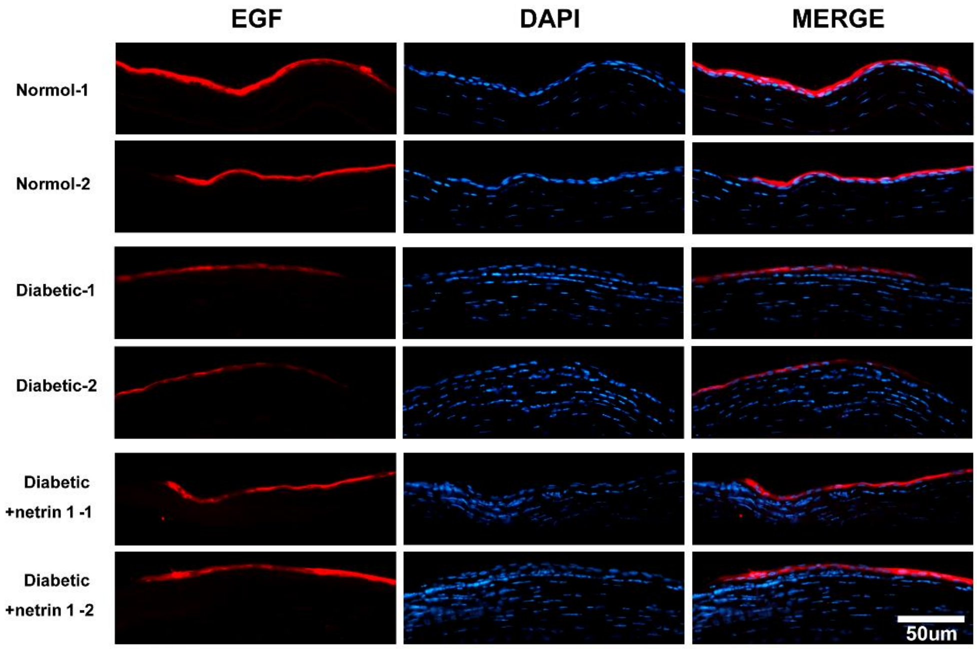

Figure S2. Netrin-1 upregulates corneal EGF expression in diabetic mice. The immunofluorescence staining results showed that the expression of EGF were decreased in diabetic mice compared to the normal mice at 48-hour post-wounded. The treatment of netrin-1 upregulated the EGF expression in diabetic mice. (3 mice per group)

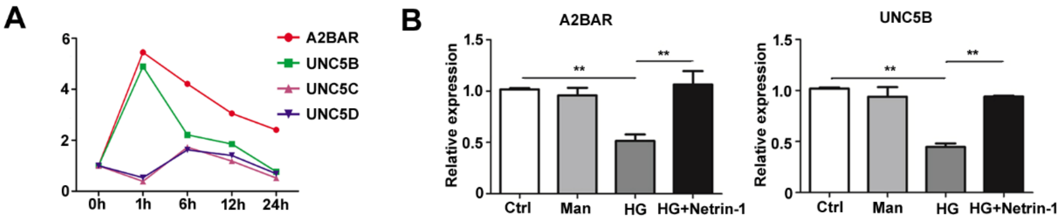

Figure S3. Netrin-1 upregulates A2BAR and UNC5B expression in high glucose-treated corneal epithelial cells. The mRNA expression of A2BAR and UNC5B were significantly up-regulated after the mouse corneal epithelial cells were wounded in normal conditions (A). Netrin-1 up-regulated the expression levels of these two receptors when compared to the high glucose-treated corneal epithelial cells and recovered the same levels as that of control or mannose osmotic control cells (B), *$p<0.05$, **$p<0.01$.
